# Health-Related Quality of Life, Comorbidities, and Lifestyle Behaviors Among Survivors of Obesity-Related and Non-Obesity-Related Cancer Types

**DOI:** 10.3390/cancers18172817

**Published:** 2026-09-01

**Authors:** William Hernández, Génesis Rodríguez-Ortiz, Lorena González-Sepúlveda, Cynthia M. Pérez, Marievelisse Soto-Salgado, Carola T. Sánchez-Díaz

**Affiliations:** 1 Department of Population and Public Health Sciences, Keck School of Medicine, University of Southern California, Los Angeles, CA 90033, USA; wh32859@usc.edu; 2 Division of Cancer Control and Population Sciences, University of Puerto Rico Comprehensive Cancer Center, San Juan, PR 00927, USA; gerodriguez@cccupr.org (G.R.-O.); lorena.gonzalez2@upr.edu (L.G.-S.); cynthia.perez1@upr.edu (C.M.P.); 3 Department of Biostatistics and Epidemiology, Graduate School of Public Health, Medical Sciences Campus, University of Puerto Rico, San Juan, PR 00936, USA; 4 Department of Social Sciences, Graduate School of Public Health, Medical Sciences Campus, University Puerto Rico, San Juan, PR 00936, USA

**Keywords:** obesity, obesity-related cancer, cancer survivors, Hispanic, quality of life, smoking, alcohol use, physical

## Abstract

**Background/Objectives**: Obesity is a well-established risk factor for several cancers, yet few studies have explored survivorship outcomes among obesity-related cancer (ORC) survivors—a growing public health concern. Beyond cancer risk, obesity is associated with poorer health-related quality of life (HRQoL), greater comorbidity burden, and adverse lifestyle behaviors during survivorship. This study examined differences in these outcomes between ORC and non-ORC survivors living in Puerto Rico. **Methods**: We analyzed 569 cancer survivors aged ≥21 years actively undergoing treatment from the Impact of Social Determinants of Health on the Cancer Care Continuum in Cancer Survivors in Puerto Rico Study, with survey data collected between 2023 and 2025. ORC status was classified based on the presence of a primary ORC (*n* = 262); all others were classified as non-ORC (*n* = 307). Outcomes included: (1) HRQoL, assessed using the Functional Assessment of Cancer Therapy-General overall score and domains; (2) comorbidities; and (3) lifestyle behaviors (i.e., physical activity, alcohol use, and smoking status). Poisson regression models with robust standard errors estimated adjusted prevalence ratios (aPRs) and 95% confidence intervals (CIs) for all outcomes except smoking status and comorbidity burden, which were analyzed using multinomial logistic regression models to estimate adjusted relative risk ratios (aRRRs). **Results**: ORC survivors had a higher relative risk of former smoking (vs. never smoking) than non-ORC survivors (aRRR: 1.74, 95% CI: 1.02–2.96). No other differences were observed between groups. Older ORC survivors reported better emotional (aPR = 0.59, 95% CI: 0.41–0.85) and physical (aPR = 0.74, 95% CI: 0.58–0.95) well-being than older non-ORC survivors. Among male participants, ORC survivors reported a lower prevalence of moderate/exceeded drinking than non-ORC survivors. **Conclusions**: Findings highlight a higher relative risk of former smoking (vs. never smoking) among ORC survivors compared with non-ORC survivors, with additional sex- and age-specific patterns in alcohol use and emotional/physical well-being.

## Simple Summary

Obesity is a well-established risk factor for 13 cancer types, whose incidence has risen over the past 20 years. Yet few studies have explored obesity associated cancers (ORC) survivors’ experiences, particularly in Hispanic/Latino communities. This study examined health-related quality of life (HRQoL), comorbidities, and lifestyle behaviors among adult cancer survivors living in Puerto Rico with an ORC compared with those who had other cancers. Using survey data from 569 participants enrolled in a study of cancer survivorship in Puerto Rico, we found that ORC survivors were, on average, older and more likely to be female. We also observed differences in smoking status between ORC and non-ORC survivors. In addition, older ORC survivors reported better emotional and physical well-being than younger survivors, and men with ORCs were less likely to report moderate or exceeded moderate alcohol use than men with non-ORCs. Further research is needed to inform culturally responsive care aimed at improving cancer survivorship among Hispanic/Latino cancer survivors.

## 1. Introduction

The CDC has identified 13 cancers strongly associated with excess body weight [1,2]. In the United States (US), 40.3% of adults are obese and are therefore at increased risk of developing ORCs [3,4]. Consistent with rising obesity prevalence, the incidence of ORCs has increased in recent decades, while the incidence of non-ORCs remained stable [5]. Given this rising burden, characterizing the health and survivorship profiles of ORC survivors is critical for identifying their distinct needs and informing targeted prevention, treatment, and survivorship care strategies.

Most survivorship research has treated cancer survivors as a homogeneous group or has focused on obesity as a risk factor, rather than distinguishing between survivors of ORCs and survivors of non-ORCs [6,7,8,9]. As a result, remains unclear whether survivors of ORCs differ from survivors of non-ORCs regarding lifestyle behaviors, comorbidities, and HRQoL, given that a cancer diagnosis compounds obesity-related risk for these same factors. This gap in knowledge is especially concerning for Hispanic/Latino populations, who experience a higher burden of obesity and related conditions in the US. Among Hispanic/Latino adults in the US, obesity prevalence is estimated at 44.8%, slightly higher than that of non-Hispanic White adults (44.2%) [10]. Moreover, adults living in Puerto Rico have an estimated prevalence of 36.2% [11], one of the highest in the US. Data from the Hispanic Community Health Study/Study of Latinos (HCHS/SOL) report that Puerto Ricans have the highest obesity incidence rate and risk for ORC among Hispanic/Latino groups [12,13]. In addition, national data suggest that ORCs are disproportionately common among Hispanic/Latino adults with metabolic syndrome [14], further underscoring the need to examine the survivorship experience of ORC survivors, given that a cancer diagnosis compounds pre-existing risk for comorbidities associated with obesity-related disease processes, particularly among Hispanic/Latino cancer survivors.

Despite the growing evidence of the detrimental impact of obesity on health outcomes among cancer survivors, virtually no data are currently available on the experiences of survivors of ORCs compared to non-obesity-related cancers (non-ORCs). Specifically, while obesity itself has been linked to diminished health-related quality of life (HRQoL) and a higher prevalence of comorbidities [8,9,15,16,17], little is known about the unique health risks among survivors of ORCs [18].

The paucity of research on health outcomes during ORC survivorship is even more marked for high-risk subgroups like Hispanic/Latino survivors. Existing work has focused on the link between obesity and worse physical health and higher prevalence of comorbidities in Hispanic/Latino cancer survivors [6,19,20,21,22]; however, to date, no studies have directly compared comorbidities and HRQoL by ORC status among Hispanic/Latino survivors. For example, a multinational study recently estimated that 33.4% of cancer patients had one or more comorbidities [23]. Another study based in the US reported that 43.4% of Hispanic/Latino cancer patients had at least one comorbidity [24]. These studies included at least one ORC (colorectal cancer) but did not differentiate between ORC and non-ORC survivors in their findings. Notable for carrying the highest risk of developing ORCs of all Hispanic/Latino subgroups, Puerto Ricans also navigate unique social and structural conditions including high levels of poverty, a fragile healthcare infrastructure, and longstanding economic constraints that influence their health outcomes [25]. In a recent study of Puerto Rican women with gynecologic cancers, 90.1% reported at least one comorbidity, 39.5% reported poor general physical health, and 33.9% experienced frequent physical distress [26]. Notably, the majority (75.1%) of these cancers were ORCs (endometrial or ovarian cancers), yet no comparisons were made with survivors of non-ORCs. To date, no studies have directly compared comorbidities and HRQoL between survivors of ORC and non-ORC in Puerto Rico.

These patterns highlight the importance of assessing health outcomes among cancer survivors living in Puerto Rico, where cancer survivors navigate a unique healthcare and sociocultural context that may influence survivorship outcomes, face elevated chronic disease risk [25], and may benefit from tailored survivorship interventions. Understanding differences between ORC and non-ORC survivors is therefore essential not only for developing culturally responsive survivorship strategies that address behavioral and clinical needs, but also for generating hypotheses to guide future research into the mechanisms underlying these differences.

## 2. Methods

### 2.1. Data Source

This cross-sectional study analyzed data from 871 eligible participants enrolled in the Impact of Social Determinants of Health on the Cancer Care Continuum in Cancer Survivors in Puerto Rico (START-PR) project, conducted between November 2023 and August 2025 [27]. The primary aim of START-PR is to evaluate the association between social determinants of health and access to care across the cancer care continuum. All participants were informed about study procedures before enrollment in accordance with protocols approved by the University of Puerto Rico Comprehensive Cancer Center Institutional Review Board (UPRCCC IRB; protocol #2411004025). They completed an anonymous 15–20 min questionnaire designed to collect information on social, clinical, and demographic characteristics. The survey, administered in person or online, was available in both Spanish and English; in-person participants were able to elect either tablet- or paper-based versions.

Eligible participants were adults aged ≥21 years residing in Puerto Rico who had received cancer treatment within the past 12 months. For this study, participants with a primary cancer diagnosis were eligible. Multiple recruitment strategies were used, including in-person study promotion by trained research assistants in collaborating oncology clinics in the San Juan Metropolitan area; outreach efforts during community health activities and professional meetings; social media; referrals from providers; and flyers in waiting rooms. For this secondary data analysis, we excluded participants with missing information on sex and age (*n* = 7), inconclusive cancer type (*n* = 26), missing cancer type (*n* = 16), and multiple cancer types (*n* = 250).

Among participants with breast cancer, those with missing age at diagnosis were excluded because they could not be classified as ORC or non-ORC (*n* = 4). The final analytic sample comprised 569 participants. Figure S1 displays the flow diagram of analytic sample derivation. This study is a secondary analysis of an existing cohort with a fixed sample size; accordingly, no a priori power calculation was performed, and we report 95% confidence intervals for all effect estimates [28].

### 2.2. Measures

#### 2.2.1. Obesity-Related Cancer

Participants were classified as having an obesity-related cancer (ORC) if their primary tumor site corresponded to one of the 13 cancer types that the CDC identifies as being associated with obesity. These include postmenopausal breast, colorectal, endometrial, esophageal adenocarcinoma, gallbladder, kidney, liver, ovarian, pancreatic, stomach, thyroid, multiple myeloma, and meningioma [1,2]. All other cancer types were classified as non-ORCs. Table S1 presents the distribution of individual cancer types among participants with ORCs and non-ORCs.

#### 2.2.2. HRQoL and HRQoL Sub-Scales

HRQoL was assessed using the Functional Assessment of Cancer Therapy-General (FACT-G) instrument [29], a validated 27-item questionnaire designed to measure four domains of HRQoL in cancer patients: Physical Well-Being (PWB), Social/Family Well-Being (SWB), Emotional Well-Being (EWB), and Functional Well-Being (FWB). Items are scored on a 5-point Likert scale ranging from 0 to 4, with higher scores indicating better well-being. Reverse-coded items were recoded according to instrument guidelines before subscale scoring [29]. Participants were categorized as having low versus high HRQoL based on the total FACT-G score. A score ≤ 70 was classified as having low HRQoL. Subscale-specific thresholds were used to identify low well-being in each domain: PWB ≤ 18, SWB ≤ 19, EWB ≤ 15, and FWB ≤ 14. Scores above these thresholds were classified as high. These thresholds correspond to the group-level cutoff scores established by Pearman et al. in a large adult cancer sample, derived as one-half standard deviation below the mean [30].

#### 2.2.3. Comorbidities

Comorbidities were grouped according to chronic diseases commonly assessed in national surveys of adult cancer survivors [31]. Participants were classified into four comorbidity burden categories: no chronic conditions (0), one chronic condition (1), two chronic conditions (2), or three or more chronic conditions (3+) based on self-report of the following: hypertension, diabetes, stroke, heart disease, chronic obstructive pulmonary disease, asthma, kidney disease, liver disease, hepatitis, arthritis. Diabetes and hypertension were also presented individually, as these cardiometabolic comorbidities align with the framework described by Maras et al. (2023) [24].

#### 2.2.4. Lifestyle Behaviors

Lifestyle behaviors included self-reported alcohol use, physical activity, and smoking. Alcohol use was categorized as no drinking or moderate/exceeded moderate drinking according to the CDC definition of moderate alcohol use using sex-specific thresholds for average drinks consumed per drinking day and consistent with the American Cancer Society Nutrition and Physical Activity Guideline for Cancer Survivors [32,33]. The moderate and exceeded moderate drinking categories were combined because of the limited sample size in the exceeded moderate drinking group. Physical activity referred to any exercise or movement outside of regular work during the past 30 days. Smoking status was classified according to CDC definitions as never, former, or current smoker based on lifetime cigarette consumption (≥100 cigarettes) and current smoking status [34].

#### 2.2.5. Covariates

Sociodemographic variables included age at cancer diagnosis (in years), sex assigned at birth (female or male), education level (less than high school, high school/some college/associate degree, or bachelor’s degree/higher), annual household income (<$20,000, $20,000–$40,000, >$40,000, or do not know/refused to report), marital status (married/living with partner or divorced/separated/widowed/single), birthplace (United States, Puerto Rico, other), self-reported residential area (urban/suburban or rural), health insurance type (private, Medicare/Medicare-Medicaid, or public), access to health care (yes/no), cancer stage (localized, regional, distant, unknown), and current cancer treatment (yes/no). Menopausal status at the time of breast cancer diagnosis was approximated using age at diagnosis, consistent with previous studies [35,36]. Participants diagnosed after age 50 were classified as having postmenopausal breast cancer, whereas those diagnosed at age 50 years or younger were classified as having premenopausal breast cancer. Covariates were selected a priori based on prior research examining social determinants of health [6,22,27] survivorship outcomes [8,9,15,16,17] and chronic disease [19,20,21] among cancer survivors.

### 2.3. Statistical Analysis

Descriptive statistics were used to summarize participant characteristics. Chi-square tests compared categorical variables, and independent *t*-tests assessed differences in continuous variables between ORC and non-ORC groups. Poisson regression models with robust standard errors estimated adjusted prevalence ratios (aPRs) and 95% confidence intervals (CIs) for associations between ORC status and each outcome, including lifestyle behaviors (physical activity and alcohol use), HRQoL total and subscale scores. Smoking status (never, former, and current) and comorbidity burden (0, 1, 2, and 3+ comorbidities) were evaluated using multinomial logistic regression models to estimate adjusted relative risk ratios (aRRRs) and 95% CIs.

All models included a prespecified set of covariates including sex, age at diagnosis, education, and cancer stage, selected a priori based on prior research and clinical relevance [6,8,9,15,16,17,19,20,21,22,27]. Additional outcome-specific covariates were selected using stepwise regression with an entry criterion of *p*-value ≤ 0.05. This approach was used to develop parsimonious adjusted models for each outcome. Specifically, for smoking status, alcohol use was included; for alcohol use, comorbidity burden, smoking status, cancer treatment, income, health insurance, and birthplace were included. For physical activity, marital status, comorbidity burden, and income were included. For overall HRQoL, comorbidity burden and birthplace were included. For FWB and PWB, physical activity, comorbidity burden, and birthplace were added. For EWB, birthplace was included; and for SWB, comorbidity burden and marital status were included. For comorbidity burden, physical activity, marital status, HRQoL, and income were included.

Interaction terms between ORC status and age and ORC status and sex were evaluated using Wald tests. Where significant, the model was stratified and estimated within each stratum. Statistical significance was defined as a two-sided *p*-value < 0.05. All analyses were conducted using Stata/SE version 18 [37].

## 3. Results

### 3.1. Sample Demographics

Table 1 describes the 569 participants; 262 (46.05%) were diagnosed with ORCs and 307 (53.95%) with non-ORCs. ORC survivors were significantly older at cancer diagnosis (mean age: 59.29 ± 12.86 years) compared to non-ORC survivors (54.34 ± 14.99 years; *p* < 0.001). A higher proportion of women were diagnosed with ORCs (80.92%) than with non-ORCs (55.63%; *p* < 0.001). ORC survivors were more likely to be born in the US (11.45%) compared to non-ORC survivors (5.45%; *p* = 0.04). No significant differences were observed between groups for educational attainment, household income, marital status, physical activity, smoking status, HRQoL total and subscale scores, number of comorbidities, or active treatment (*p* > 0.05 for all).

### 3.2. Multivariable Analyses

Multinomial logistic regression models indicated that ORC survivors had a significantly higher relative likelihood of reporting being former smokers relative to never smoking compared with non-ORC survivors (aRRR: 1.74, 95% CI: 1.02–2.96; Table 2) after adjusting for sex, education, age at diagnosis, cancer stage, and alcohol use. No significant association between ORC status and current smoking relative to never smoking was observed (aRRR: 1.09, 95% CI: 0.41–2.91). No significant differences by ORC status were observed for physical activity, alcohol use, or HRQoL total and subscale scores.

### 3.3. Interaction Analyses

**Figure 1:**
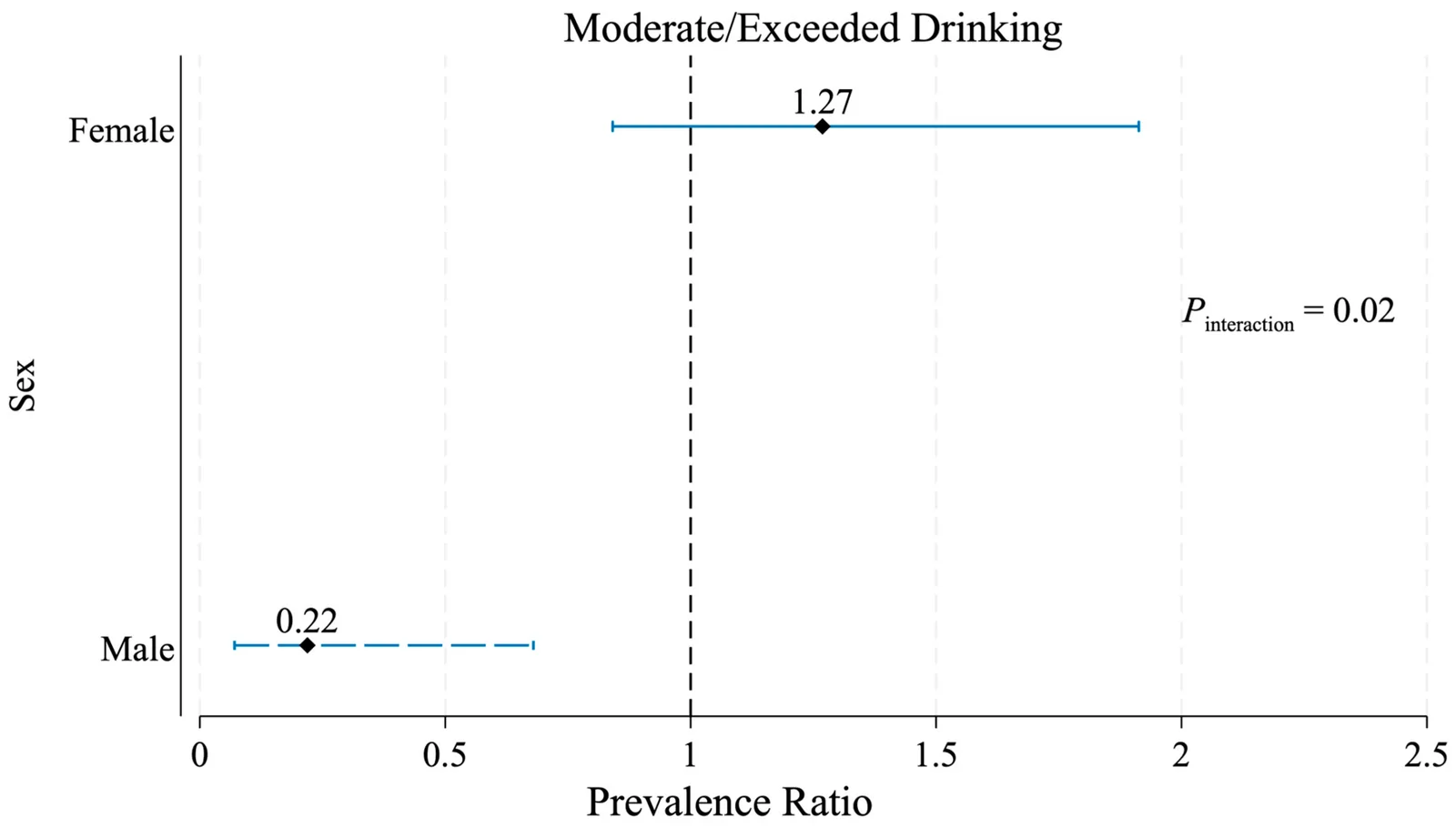
Stratified analyses and interaction tests of the association between ORC status and moderate/exceeded drinking (vs. no drinking). The vertical dashed line indicates an aPR of 1.00. Solid and long-dashed lines denote females and males, respectively. Models were adjusted for sex, education, age at diagnosis, cancer stage, comorbidity burden, smoking status, cancer treatment, income, insurance, and birthplace. Stratum sample sizes were females (*n* = 392) and males (*n* = 177).

The association between ORC status and alcohol use varied by sex (*p* = 0.02; Figure 1). Among males, ORC survivors had a significantly lower prevalence of moderate/exceeded drinking than non-ORC survivors (aPR = 0.22, 95% CI: 0.07–0.68). In contrast, among females, ORC survivors had a higher, although not statistically significant, prevalence of moderate/exceeded drinking than non-ORC survivors (aPR = 1.27, 95% CI: 0.84–1.91).

**Figure 2:**
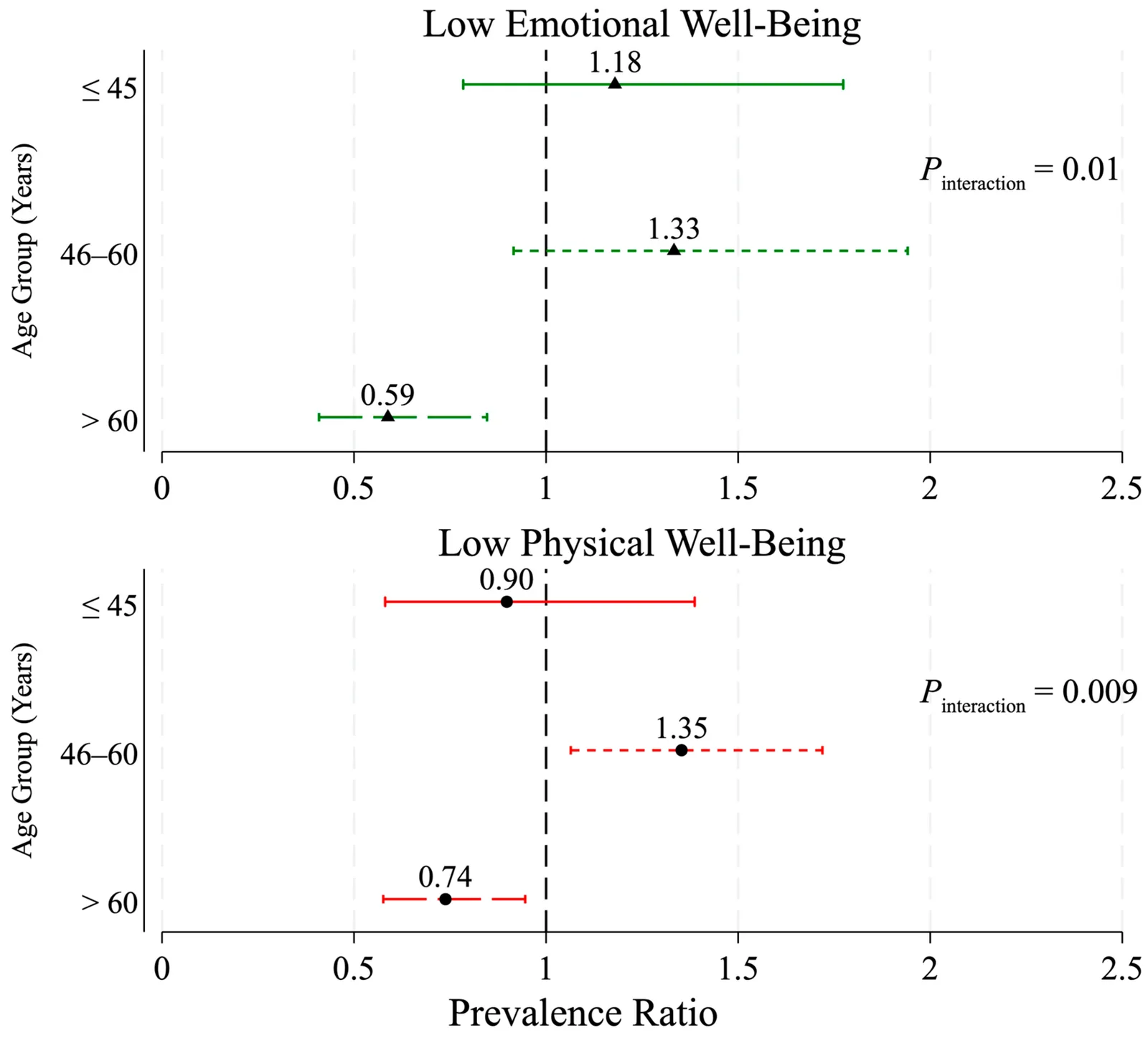
Stratified analyses and interaction tests of the associations between ORC status and low emotional well-being (vs. high emotional well-being) and low physical well-being (vs. high physical well-being). The vertical dashed line indicates an aPR of 1.00. Solid, short-dashed, and long-dashed lines denote participants aged ≤45, 46–60, and >60 years, respectively. All models were adjusted for sex, education, age at diagnosis, and cancer stage. Additional covariates included birthplace for low emotional well-being and physical activity, comorbidity burden, and birthplace for low physical well-being. Stratum sample sizes were ≤45 years (*n* = 124), 46–60 years (*n* = 196), and >60 years (*n* = 249).

Age at diagnosis also modified associations with EWB (*p* = 0.01) and PWB (*p* = 0.009; Figure 2). In age-stratified analyses, ORC survivors aged >60 years were less likely than their non-ORC counterparts to report low EWB (aPR = 0.59, 95% CI: 0.41–0.85) and low PWB (aPR = 0.74, 95% CI: 0.58–0.95). In contrast, ORC survivors aged 46–60 years had a significantly higher prevalence of low PWB (aPR = 1.35, 95% CI: 1.06–1.72), with no significant difference in EWB. No significant associations with EWB or PWB were identified among participants aged ≤45. Age-stratified linear regression analyses of HRQoL and its subdomains showed broadly similar patterns (Table S2).

## 4. Discussion

This study is among the first to characterize the relationship between ORC status and survivorship outcomes—including HRQoL, comorbidities, and lifestyle behaviors—among cancer survivors living in Puerto Rico, addressing a critical gap in the literature on Hispanic/Latino populations. Overall, most outcomes examined did not differ significantly by ORC status, suggesting that ORC and non-ORC survivors share largely similar survivorship profiles in this population. Where significant differences did emerge, findings highlight a higher relative risk of former smoking (vs. never smoking) and a lower prevalence of moderate/exceeded drinking among ORC survivors compared with non-ORC survivors, along with additional sex- and age-specific patterns in alcohol use and emotional/physical well-being. Together, these findings suggest that behavioral risk factors, rather than comorbidities, represent the primary axis of difference between ORC and non-ORC survivors in this population, and may be particularly relevant targets for tailored survivorship care. Our findings align with prior research documenting associations between obesity and smoking among cancer survivors [38,39]. We observed a higher relative likelihood of reporting being a former smoker (vs. never smoking) among ORC survivors compared with non-ORC survivors (aRRR: 1.74, 95% CI: 1.02–2.96) [40,41]. A potential explanation for this pattern is that a cancer diagnosis often serves as a “teachable moment,” prompting increased motivation for health behavior change, including smoking cessation [42,43]; ORC survivors, whose treatment and survivorship care may place greater emphasis on lifestyle counseling to address modifiable cardiometabolic and recurrence risk factors, may be particularly likely to act on this opportunity to quit. No significant interactions were observed by age or sex, suggesting that this pattern is consistent across demographic groups in our sample. Further research is needed to understand the factors underlying this more favorable cessation pattern among ORC survivors, and whether they could be leveraged to support cessation efforts among non-ORC survivors as well.

Prior studies among cancer survivors have generally examined obesity itself, rather than ORC status, as a predictor of HRQoL, leaving the survivorship experience specific to ORC survivors comparatively understudied [9,44]. To address this, we examined whether HRQoL differed by ORC status in our sample of cancer survivors living in Puerto Rico. We found ORC survivors aged >60 years were significantly less likely to report low PWB and EWB, whereas those aged 46–60 years reported a higher prevalence of low PWB. These exploratory findings suggest that the relationship between ORC status and HRQoL may vary across the life course [45,46]. Although not directly evaluated in this study, two complementary hypotheses may help explain this pattern. Differences in EWB may reflect age-related shifts in emotion regulation: consistent with socioemotional selectivity theory, as time horizons narrow with age, older adults increasingly prioritize emotionally meaningful goals and interactions, which has been associated with better emotional well-being relative to younger and middle-aged adults [47]. Differences in PWB may partly reflect response shift, whereby survivors recalibrate their internal standards for physical health over time, such that comparable symptoms are rated as less burdensome with age or longer time since diagnosis [48]. Additional studies incorporating validated measures of emotion regulation and response shift in ORC survivors are warranted. If confirmed in future studies, these findings may have implications for lifestyle-focused interventions, including physical activity promotion and nutritional counseling.

We found no significant differences in physical activity by ORC status. However, our measure captured any exercise or movement outside of work during the past 30 days and did not assess whether participants met recommended levels of physical activity. This likely reflects the overall low physical activity observed in the sample, consistent with population-level estimates in Puerto Rico [49]. Environmental and structural barriers, such as limited access to safe recreational spaces, walkable neighborhoods, and community facilities, may play a larger role in shaping physical activity than ORC status [49,50]. Taken together with population-level evidence from Puerto Rico, these findings highlight the importance of continuing to understand physical activity patterns among cancer survivors. Future studies using guideline-based measures of physical activity are needed to better characterize differences by ORC status [19,24,51].

Exploratory analyses further identified a significant interaction by sex, indicating that this association was observed among males but not among females. Among males, ORC survivors had a lower prevalence of moderate/exceeded drinking than non-ORC survivors, whereas no significant differences were observed among females. Prior research using All of Us data found that males were more likely to engage in risky drinking behaviors, including binge and hazardous drinking in the past year [52] and that male cancer survivors had higher odds of current and binge drinking in the past year, with alcohol use varying by cancer types [53]. However, these studies did not examine whether the association between cancer type and alcohol use differed by sex. Sex-related factors may also contribute to these patterns, as men generally consume more alcohol and engage in heavier drinking than women, leaving greater room for behavioral change following a cancer diagnosis. In contrast, women tend to drink less and experience stronger social pressures to limit alcohol use, potentially resulting in less behavioral variation to detect differences by ORC status [54]. If confirmed in future studies, these findings may have implications for sex-specific behavioral counseling within survivorship care, particularly with respect to alcohol use among male survivors.

This study contributes to the cancer survivorship literature by addressing a critical gap in understanding ORC survivorship within Hispanic/Latino populations. However, several limitations should be considered. First, we could not adjust for obesity because obesity status at diagnosis was unavailable. Although obesity status at diagnosis would have allowed us to evaluate the independent role of obesity, the objective of this exploratory secondary analysis was to characterize survivorship profiles according to ORC status among cancer survivors in Puerto Rico. Future studies should incorporate obesity status at diagnosis to further investigate the relationship between obesity, ORC status, and health-related outcomes. Second, the exclusion of participants with multiple cancer diagnoses (29% of the eligible sample) may have introduced selection bias. Third, behavioral and comorbidity measures were self-reported, which may have introduced social desirability bias for behavioral variables such as alcohol use and smoking status, while comorbidity reporting may have been affected by recall bias. In addition, menopausal status was approximated using age at diagnosis (≥50 years), an imperfect proxy that may misclassify women whose natural menopause occurred earlier or later. However, this approximation is consistent with the average age at natural menopause among Puerto Rican women [36]. Finally, recruitment through clinical settings may overrepresent survivors who are more engaged with healthcare, potentially limiting generalizability to survivors less engaged in care. Despite these limitations, our study had several strengths, including the use of validated measures such as the FACT-G to assess HRQoL. Additionally, we adjusted for covariates informed by prior research on the social determinants of health among cancer survivors [6,8,9,15,16,17,19,22,27]. Our study is among the first to compare HRQoL, lifestyle behaviors, and comorbidities between ORC and non-ORC survivors in Puerto Rico, addressing an important gap in the literature.

Future research should incorporate prospective longitudinal designs with repeated assessments of HRQoL, comorbidities, and lifestyle behaviors, integrating objective adiposity measures and cancer registry linkages to more accurately characterize the association between ORC status and these outcomes over time.

## 5. Conclusions

In conclusion, ORC status was associated with smoking status, but not with overall HRQoL, physical activity, or comorbidities. Exploratory analyses suggested heterogeneity by age and sex, underscoring the need for tailored survivorship care strategies. These findings support public health efforts to characterize health outcomes unique to the ORC survivorship experience. Survivorship experiences in this group are shaped not only by obesity and cancer type, but also by age, sex, and broader structural factors associated with health. Considering these factors, there is a particular need to more accurately characterize ORC survivors living in Puerto Rico. Improving our understanding of ORC survivors in Puerto Rico is essential for informing clinicians, guiding culturally responsive follow-up care, and supporting policymakers and community programs in developing interventions that address the unique needs of this population. Such efforts are critical for reducing differences in cancer outcomes across Hispanic/Latino communities.

## Figures and Tables

**Table 1:** Characteristics of participants in the START-PR study (November 2023–August 2025) by ORC status (*n* = 569).

		ORC		Non-ORC		
		*n =* 262		*n =* 307		
Characteristics	Total *n* *n* = 569	*n*	%	*n*	%	*p*-Value ^a^
Current age, mean (SD)		61.16 (12.72)		56.60 (14.59)		<0.001
Age group						
≤45	124	34	12.98	90	29.34	<0.001
46–60	196	104	39.69	92	29.97	
>60	249	124	47.33	125	40.72	
Age at cancer diagnosis, mean (SD)		59.29 (12.86)		54.34 (14.99)		<0.001
Sex						<0.001
Female	392	212	80.92	180	55.63	
Male	177	50	19.08	127	41.37	
Education						0.27
Less than high school	78	31	11.97	47	15.51	
High school/Some college/Associate degree	244	109	42.08	135	44.55	
At least a bachelor’s degree	240	119	45.95	121	39.93	
Household income (USD)						0.81
≤$19,999	227	106	42.23	121	40.47	
$20,000-$39,999	129	60	23.90	69	23.08	
≥$40,000	106	49	19.52	57	19.06	
Don’t know/Refused to report	88	36	14.34	52	17.39	
Marital status						0.18
Married/living with partner	305	133	51.15	172	56.77	
Divorced/Separated/Widowed/ Single	258	127	48.85	131	43.23	
Birthplace						0.04
United States	47	30	11.45	17	5.45	
Puerto Rico	483	217	82.82	266	87.50	
Other	36	15	5.73	21	6.73	
Residential area						0.32
Rural	175	75	29.07	100	33.00	
Urban/Suburban	386	183	70.93	203	67.00	
Health insurance						0.46
Private	159	77	29.62	82	26.97	
Medicare or Medicare and Medicaid	248	107	41.15	141	46.38	
Public	157	76	29.23	81	26.64	
Cancer Stage						0.16
Localized	359	162	62.55	197	65.23	
Regional	102	55	21.24	47	15.56	
Distant	42	21	8.11	21	6.95	
Unknown	58	21	8.11	37	12.25	
Current cancer treatment						0.95
Yes	489	226	86.26	263	85.95	
No	79	36	13.74	43	14.05	
Postmenopausal status ^b^						<0.001
Yes	248	174	83.65	74	41.57	
No	138	34	16.35	104	58.43	
Physical activity (last 30 days)						0.17
Yes	188	79	30.38	109	35.86	
No	376	181	69.62	195	64.14	
Smoking Status						0.53
Never	445	200	79.05	245	80.86	
Former	90	45	17.79	45	14.85	
Current	21	8	3.16	13	4.29	
Alcohol use (last 30 days)						0.43
Moderate/Exceed Moderate	117	50	19.46	67	22.19	
No	442	207	80.52	235	77.81	
Comorbidity burden ^c^						0.55
0	178	75	42.13	103	57.87	
1	156	78	50.00	78	50.00	
2	117	54	46.15	63	53.85	
3+	118	55	46.61	63	53.39	
Diabetes						0.99
Yes	137	63	11.07	74	13.01	
No	419	193	33.92	226	39.72	
Hypertension						0.46
Yes	249	119	20.91	130	22.85	
No	307	137	24.08	170	29.88	
Overall HRQoL ^d^						0.95
Mean (SD) (Range: 9–108)		70.01 (19.24)		70.99 (19.66)		0.28
Low (≤70)	260	120	45.80	140	45.60	
Normal/High (71+)	309	142	54.20	167	54.40	
Physical Well-Being (PWB)						0.34
Mean (SD) (Range: 0–28)		16.60 (7.37)		17.18 (7.11)		0.28
Low (≤18)	308	147	56.11	161	52.44	
Normal/High (19+)	261	115	43.89	146	47.56	
Social/Family Well-Being (SWB)						0.50
Mean (SD) (Range: 0–28)		20.06 (5.71)		20.27 (6.48)		0.18
Low (≤19)	216	103	39.31	113	36.81	
Normal/High (20+)	353	159	60.69	194	63.19	
Emotional Well-Being (EWB)						0.71
Mean (SD) (Range: 0–24)		16.22 (5.36)		16.57 (5.55)		0.22
Low (≤15)	219	103	39.31	116	37.79	
Normal/High (16+)	350	159	60.69	191	62.21	
Functional Well-Being (FWB)						0.77
Mean (SD) (Range: 0–28)		17.12 (6.67)		17.03 (6.38)		0.57
Low (≤14)	192	90	34.35	102	32.22	
Normal/High (15+)	377	172	65.65	205	66.78	

^a^ *p*-values were calculated using *t*-tests for continuous variables and chi-square tests for categorical variables. ^b^ Menopause status approximated using age at diagnosis: >50 classified as postmenopausal; ≤50 as premenopausal. ^c^ Comorbidities include: hypertension, diabetes, stroke, heart disease, chronic obstructive pulmonary disease, asthma, kidney disease, liver disease, hepatitis, arthritis. ^d^ Thresholds for clinical significance: FACT-G ≤ 70; FWB ≤ 14; PWB ≤ 18; EWB ≤ 15; SWB ≤ 19. ORC: obesity-related cancer, Non-ORC: non-obesity-related cancer, SD: standard deviation, FACT-G: Functional Assessment of Cancer Therapy-General.

**Table 2:** Prevalence ratios for patient-reported outcomes and lifestyle behaviors by ORC status, with non-ORC as the reference group (*n* = 569).

Lifestyle Behaviors and Outcomes	Effect Estimate (95% CI) ^a^
Smoking Status ^b^	
Never	1.00
Former	1.74 (1.02–2.96)
Current	1.09 (0.41–2.91)
Lack of Physical Activity in the Last 30 days (Yes/No) ^c^	1.07 (0.95–1.22)
Alcohol Use in the Last 30 days ^d^	
No	1.00
Moderate/exceeded drinking	0.90 (0.64–1.27)
Comorbidity burden ^e^	
0	1.00
1	1.18 (0.72–1.93)
2	1.06 (0.61–1.84)
3+	0.87 (0.50–1.52)
Overall HRQoL (Low/High) ^f^	1.02 (0.84–1.24)
Physical Well-Being (Low/High) ^g^	1.01 (0.87–1.18)
Social/Family Well-Being (Low/High) ^h^	1.04 (0.84–1.30)
Emotional Well-Being (Low/High) ^i^	1.03 (0.82–1.29)
Functional Well-Being (Low/High) ^j^	1.04 (0.81–1.33)

^a^ Prevalence ratios (aPRs) and 95% CIs for binary outcomes were estimated using modified Poisson regression with robust standard errors. Relative risk ratios (aRRRs) and 95% CIs for the multi-level outcomes were estimated using multinomial logistic regression. Each model was adjusted for sex, education, age at diagnosis, and cancer stage. Additional covariates were selected using stepwise regression and varied by outcome. Covariates specific to each model are indicated by superscript letters as follows: ^b^ The model was additionally adjusted for alcohol use. ^c^ Marital status, comorbidity burden, income; ^d^ comorbidity burden, smoking status, cancer treatment, income, insurance, and birthplace; ^e^ overall HRQoL, physical activity, income, and marital status; ^f^ comorbidity burden and birthplace; ^g^ physical activity, comorbidity burden, and birthplace; ^h^ comorbidity burden, marital status; ^i^ birthplace; ^j^ physical activity, comorbidity burden and birthplace.

## Data Availability

The data supporting this study are available from M.S.-S. upon request.

## Supplementary Materials

The following supporting information can be downloaded at: https://www.mdpi.com/article/10.3390/cancers18172817/s1, Figure S1. Flow diagram of analytic sample derivation. Table S1. Distribution of ORC and non-ORC types. Table S2. Linear regression of HRQoL and subdomains by ORC status, stratified by age.

## Abbreviations

The following abbreviations are used in this manuscript:

ORC	Obesity-Related Cancer
US	United States
START-PR	Impact of Social Determinants of Health on the Cancer Care Continuum in Cancer Survivors in Puerto Rico
HRQoL	Health-Related Quality of Life
FACT-G	Functional Assessment of Cancer Therapy-General
aPR	Adjusted Prevalence Ratio
CI	Confidence Interval
CDC	Centers for Disease Control and Prevention
HCHS/SOL	Hispanic Community Health Study/Study of Latinos
UPRCCC	University of Puerto Rico Comprehensive Cancer Center
IRB	Institutional Review Board
PWB	Physical Well-Being
SWB	Social/Family Well-Being
EWB	Emotional Well-Being
FWB	Functional Well-Being
GOPA!	Global Observatory for Physical Activity
NCI	National Cancer Institute
CAPAC	Cancer Prevention and Control Program
CRTEC	Cancer Research Training and Education Coordination
NIGMS	National Institute of General Medical Sciences
