# Supplementary material for: Health-Related Quality of Life, Comorbidities, and Lifestyle Behaviors Among Survivors of Obesity-Related and Non-Obesity-Related Cancer Types
Source: Cancers (Basel). 2026 Sep 1;18(17):2817. doi: 10.3390/cancers18172817 (PMC13565206; doi:10.3390/cancers18172817)
Supplement: Supplementary file 1 [file cancers-18-02817-s001.zip › Supplementary File.pdf]

**Supplementary File**

Supplementary Figure S1. Flow diagram of analytic sample derivation…………………………..2

Supplementary Table S1. Distribution of ORC and non-ORC types..............................................3

Supplementary Table S2. Linear regression of HRQoL and subdomains by ORC status, stratified by age………………………………………….………………...…………………..4

Figure S1. Flow diagram of analytic sample derivation.

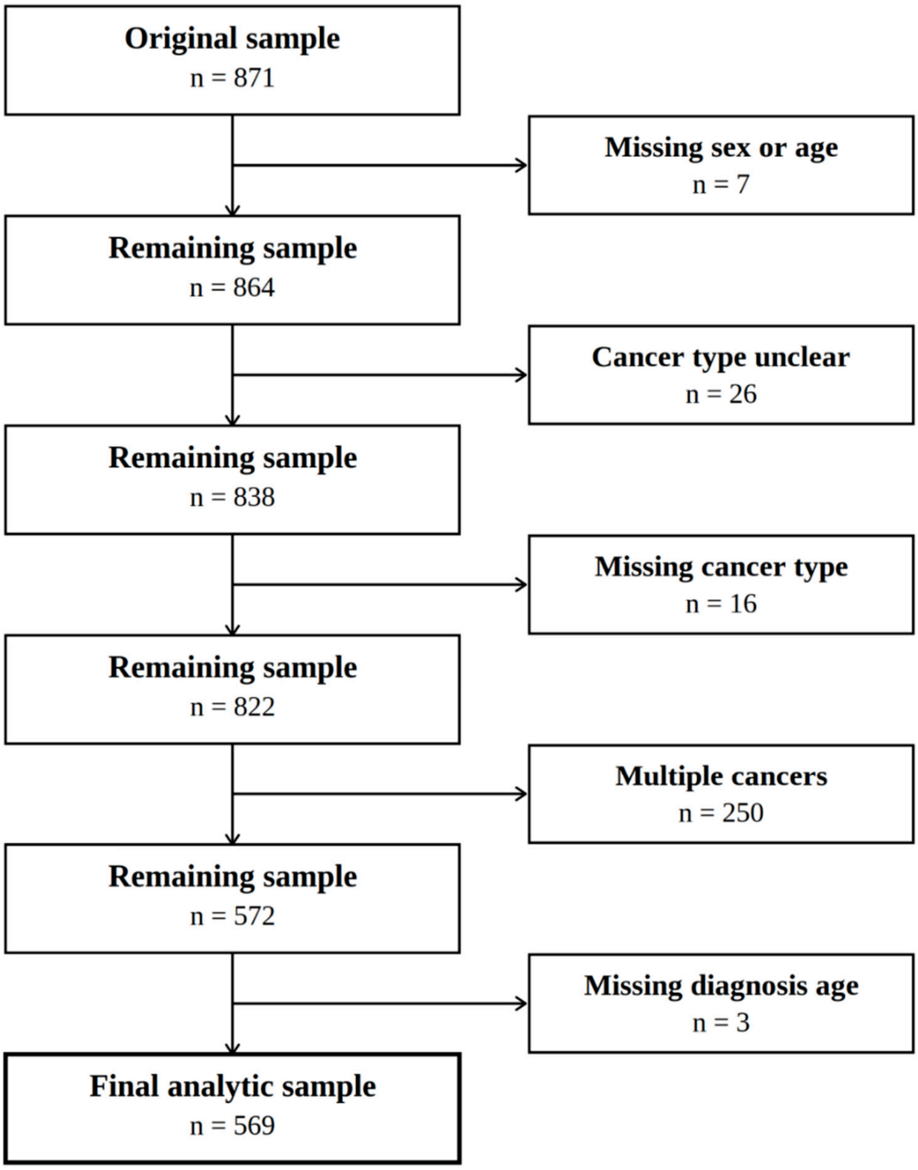

Supplementary Table S1. Distribution of ORC and non-ORC types.

| Cancer type | n | % |
|---|---|---|
| **Obesity-related cancers (ORC)** | **262** | **100.00** |
| Postmenopausal breast cancer | 104 | 39.69 |
| Colon and rectum | 44 | 16.79 |
| Ovary | 27 | 10.31 |
| Uterine corpus | 24 | 9.16 |
| Multiple myeloma | 16 | 6.11 |
| Pancreas | 13 | 4.96 |
| Thyroid | 12 | 4.58 |
| Liver and bile duct | 6 | 2.29 |
| Kidney and renal pelvis | 5 | 1.91 |
| Stomach | 5 | 1.91 |
| Esophagus | 5 | 1.91 |
| **Non-obesity-related cancers (non-ORC)** | **307** | **100.00** |
| Premenopausal breast cancer | 67 | 21.82 |
| Prostate | 49 | 15.96 |
| Cervix | 37 | 12.05 |
| Leukemia | 29 | 9.45 |
| Non-Hodgkin lymphoma | 25 | 8.14 |
| Brain and other nervous system | 22 | 7.17 |
| Head and neck | 16 | 5.21 |
| Lung and bronchus | 15 | 4.89 |
| Urinary bladder | 7 | 2.28 |
| Testis | 4 | 1.30 |
| Bone marrow | 3 | 0.98 |
| Other | 33 | 10.75 |

Supplementary Table S2: Linear regression of HRQoL and subdomains by ORC status, stratified by age.

| | FACT-G total score[a] | Physical well-being[b] | Functional well-being[b] | Social well-being[c] | Emotional well-being[d] |
|---|---|---|---|---|---|
| | **β (95% CI)** | **β (95% CI)** | **β (95% CI)** | **β (95% CI)** | **β (95% CI)** |
| **ORC Status** | | | | | |
| Non-ORC | ref | ref | ref | ref | ref |
| ORC | -0.59 (-3.96, 2.79) | -0.24 (-1.46, 0.99) | 0.22 (-0.90, 1.34) | 0.00 (-1.04, 1.04) | -0.32 (-1.30, 0.66) |
| **Age-stratified association** | | | | | |
| ≤ 45 years | -0.23 (-7.98, 7.52) | -0.61 (-3.66, 2.44) | 0.75 (-1.96, 3.46) | 1.86 (-0.78, 4.50) | -1.89 (-4.12, 0.33) |
| 46-60 years | -4.24 (-10.08, 1.61) | -1.89 (-3.87, 0.09) | -1.06 (-2.97, 0.85) | 0.03 (-1.58, 1.64) | -1.38 (-3.04, 0.27) |
| > 60 years | 4.72 (-0.17, 9.61) | **2.35 (0.47, 4.23)** | 0.97 (-0.69, 2.63) | -0.56 (-2.25, 1.13) | **2.35 (0.90, 3.80)** |
| **P for interaction** | 0.18 | **0.02** | 0.24 | 0.12 | **0.001** |

Each model was adjusted for sex, education, age at diagnosis, and cancer stage. Additional covariates were selected using stepwise regression and varied by outcome. Outcome-specific covariates are indicated by superscript letters as follows: [a]comorbidity burden and birthplace; [b]physical activity, comorbidity burden, and birthplace; [c]comorbidity burden and maritial status; [d]birthplace. β coefficients represent adjusted mean differences comparing ORC with non-ORC survivors. Age-stratified estimates represent adjusted mean differences within each age group. P values correspond to the ORC × age group interaction.
